# The Pyroptosis-Related Gene Signature Predicts the Prognosis of Hepatocellular Carcinoma

**DOI:** 10.3389/fmolb.2021.781427

**Published:** 2022-01-03

**Authors:** Shuqiao Zhang, Xinyu Li, Xiang Zhang, Shijun Zhang, Chunzhi Tang, Weihong Kuang

**Affiliations:** ^1^ First Affiliated Hospital of Chinese Medicine, Guangzhou University of Chinese Medicine, Guangzhou, China; ^2^ Medical College of Acupuncture-Moxibustion and Rehabilitation, Guangzhou University of Chinese Medicine, Guangzhou, China; ^3^ The Second Clinical Medical College, Zhejiang Chinese Medical University, Hangzhou, China; ^4^ Department of Traditional Chinese Medicine, The First Affiliated Hospital, Sun Yat-sen University, Guangzhou, China; ^5^ Guangdong Key Laboratory for Research and Development of Natural Drugs, School of Pharmacy, Guangdong Medical University, Dongguan, China

**Keywords:** hepatocellular carcinoma, pyroptosis, prognosis, immune infiltration, signature

## Abstract

**Objective:** Hepatocellular carcinoma (HCC) is a genetically and phenotypically heterogeneous tumor, and the prediction of its prognosis remains a challenge. In the past decade, studies elucidating the mechanisms that induce tumor cell pyroptosis has rapidly increased. The elucidation of their mechanisms is essential for the clinical development optimal application of anti-hepatocellular carcinoma therapeutics.

**Methods:** Based on the different expression profiles of pyroptosis-related genes in HCC, we constructed a LASSO Cox regression pyroptosis-related genes signature that could more accurately predict the prognosis of HCC patients.

**Results:** We identified seven pyroptosis-related genes signature (*BAK1*, *CHMP4B*, *GSDMC*, *NLRP6*, *NOD2*, *PLCG1*, *SCAF11*) in predicting the prognosis of HCC patients. Kaplan Meier survival analysis showed that the pyroptosis-related high-risk gene signature was associated with poor prognosis HCC patients. Moreover, the pyroptosis-related genes signature performed well in the survival analysis and ICGC validation group. The hybrid nomogram and calibration curve further demonstrated their feasibility and accuracy for predicting the prognosis of HCC patients. Meanwhile, the evaluation revealed that our novel signature predicted the prognosis of HCC patients more accurately than traditional clinicopathological features. GSEA analysis further revealed the novel signature associated mechanisms of immunity response in high-risk groups. Moreover, analysis of immune cell subsets with relevant functions revealed significant differences in aDCs, APC co-stimulation, CCR, check-point, iDCs, Macrophages, MHC class-I, Treg, and type II INF response between high- and low-risk groups. Finally, the expression of Immune checkpoints was enhanced in high-risk group, and m6A-related modifications were expressed differently between low- and high-risk groups.

**Conclusion:** The novel pyroptosis-related genes signature can predict the prognosis of patients with HCC and insight into new cell death targeted therapies.

## Introduction

Hepatocellular carcinoma (HCC) is the third most aggressive and lethal disease, accounting for approximately 75% of liver cancer cases, and is a highly genetically and phenotypically heterogeneous malignancy with 830,000 deaths in 2020 (Petrick et al., 2016; Moon and Ro, 2021). Alcohol abuse, obesity, diabetes, and metabolic syndromes are significant risk factors for HCC progression, and inflammation caused by these risk factors promotes liver fibrosis, leading to cirrhosis and ultimately HCC(Mittal and El-Serag, 2013; Kim and Viatour, 2020). Patients with HCC are asymptomatic at the early stage, which seriously delays timely diagnosis. Patients diagnosed at the late stage of HCC are not suitable for radical surgery, resulting in minimal availability and effectiveness of therapeutic options (Llovet et al., 2008). Thus, novel biomarkers that can discriminate patients at high risk for HCC are urgently needed to improve personalized HCC prognostic prediction accuracy and treatment.

In the past decade, studies elucidating the mechanisms that induce tumor cell pyroptosis has rapidly increased (Derangere et al., 2014; Jiang et al., 2017; Wang Y et al., 2018). Pyroptosis is an inflammatory caspase-dependent cell death type characterized by pore formation, cell swelling and rupture of the plasma membrane, and release of intracellular contents (Ruan et al., 2020). Pyroptosis therapies are increasing as opportunities to inhibit cancer development. Meanwhile, pyroptosis promotes inflammatory cell death and inhibits cancer cell proliferation and migration, and decreased expression of some pyroptotic inflammasomes has been found in cancer cells (Fang et al., 2020). Apoptosis is widely studied as a major form of regulated cell death underlying tumor pathogenesis and therapy. Still, cancer-associated defects in apoptosis induction and execution contribute to a significant proportion of treatment failures (Ng et al., 2012; Holohan et al., 2013; Hata et al., 2014). The clear molecular pathways mediating necrotic types of cell death have recently been uncovered, the long-standing view of apoptosis as a standard regulating mechanism of death programs has changed (Vanden Berghe et al., 2014; Conrad et al., 2016; Wallach et al., 2016). The previously unknown mechanism of pyroptosis as a molecularly targeted pathway to eradicate oncogene addicted tumor cells may have important implications for the clinical development and optimal application of anticancer therapeutics (Lu et al., 2018).

However, studies on the functions and mechanisms of pyroptosis-related genes in HCC progression remain scarce. A systematic evaluation of pyroptosis-related gene prognostic signatures and their correlation with HCC patients may further our understanding of HCC mechanisms and provide new applications for a rapid, effective, and specific diagnosis and effective therapy.

A novel pyroptosis-related prognostic signature of differentially expressed genes in HCC was established in our study. Then we studied their role in the prognosis of HCC patients and the associated immune response and the effect of N6- methylation on adenosine (m6A) modification.

## Methods

### Data Collection

We extracted RNA sequencing (50 normal and 374 tumors) data of 377 patients from the TCGA-LIHC (https://portal.gdc.cancer.gov/repository) dataset, and RNA sequencing (273 tumors) data of 261 patients from the ICGC-LIRI-JP (https://dcc.icgc.org/releases/current/Projects/LIRI-JP) dataset. Clinical characteristics of HCC patients in the TCGA and ICGC dataset was shown in Supplementary Table S1. The corresponding pyroptosis-related genes in Supplementary Table S2 were identified from the previous studies of multiple regulatory mechanisms of pyroptosis in the tumor microenvironment (Xia et al., 2019; Shao et al., 2021; Ye et al., 2021) and Molecular Signatures database (http://www.gsea-msigdb.org/gsea/login.jsp) (Liberzon et al., 2015). Before comparison, normalization of the expression data in both datasets values was performed using fragment per kilobase million (FPKM) values. The association between pyroptosis-related genes and HCC was assessed using the “limma” R package, and the correlation was considered significant if the *p*-value was <0.05. The protein-protein interaction (PPI) network of the pyroptosis-related differentially expressed genes (DEGs) was developed by STRING (Szklarczyk et al., 2021), version 11.5 (https://string-db.org/).

### Functional Enrichment Analysis

First, the biological process (BP), cellular component (CC), and molecular function (MF) of the pyroptosis-related DEGs were investigated using Gene Ontology (GO). Then the biological pathway functions of DEGs were further analyzed by Kyoto Encyclopedia of Genes and Genomes (KEGG) based data in R software version 4.0.5.

### Development of the Pyroptosis-Related Genes Prognostic Signature

To construct an accurate and reliable prognostic prediction signature for HCC patients, we first screened the resulting pyroptosis-related DEGs for those with predictive value using univariate Cox regression analysis and then further processed using LASSO regression analysis prevent the fitting of risk models. Finally, the pyroptosis-related genes signature was constructed and stratified according to the risk score ( ${\text{e}}^{\text{sum}\left(\text{each genes' expression}\times \text{corresponing cofficient}\right)\;}$ ). Finally, HCC patients were divided into high-risk (≥median) and low-risk (<median) groups according to the median value of the risk score of the established prognostic model.

### The Predictive Nomogram and Calibration Curves

To create a clinically practical approach in predicting the 1, 3, and 5-year overall survival rate of HCC patients, we developed a hybrid nomogram model incorporating independent prognostic factor including risk score signature, gender, age, TMN, stage, and grade. We then validated the accuracy of the nomogram model for judging the prognosis situation of HCC patients using the degree of fit of the calibration curve to the actual observed values.

### Immune Profile Analysis

Meanwhile, immune cell infiltration levels of the seven pyroptosis-related genes signatutre in individual samples in two risk groups were quantified by single-sample gene set enrichment analysis (ssGSEA) (Rooney et al., 2015). The cellular immune responses of the pyroptosis-related genes signature between subgroups were then evaluated by comparing the results of CIBERSORT(Newman et al., 2015; Charoentong et al., 2017), CIBERSORT−ABS (Wang L et al., 2020), QUANTISEQ (Plattner et al., 2020), MCPCOUNTER (Shi et al., 2020), XCELL (Aran et al., 2017), EPIC(Racle et al., 2017), and TIMER (Li et al., 2017) algorithms. In addition, we evaluated differences in immune function expression by tumor-infiltrating immune cell subsets in the two risk groups. Finally, we analyzed the status of m6A methylation modification in high and low-risk groups to explore the possible impact of the seven pyroptosis-related genes on the activities of methyltransferases, demethylases, and methylated reader proteins in HCC.

### Independent Prognostic Validation of the Prognostic Signature

Information on clinical characteristics, including gender, age and staging data, of HCC patients in the TCGA dataset and HCC patients in the ICGC dataset was extracted. These clinical variables in combination with our risk score prognostic signature was analyzed by univariate and multivariate Cox regression.

### Statistical Analysis

**FIGURE 1:**
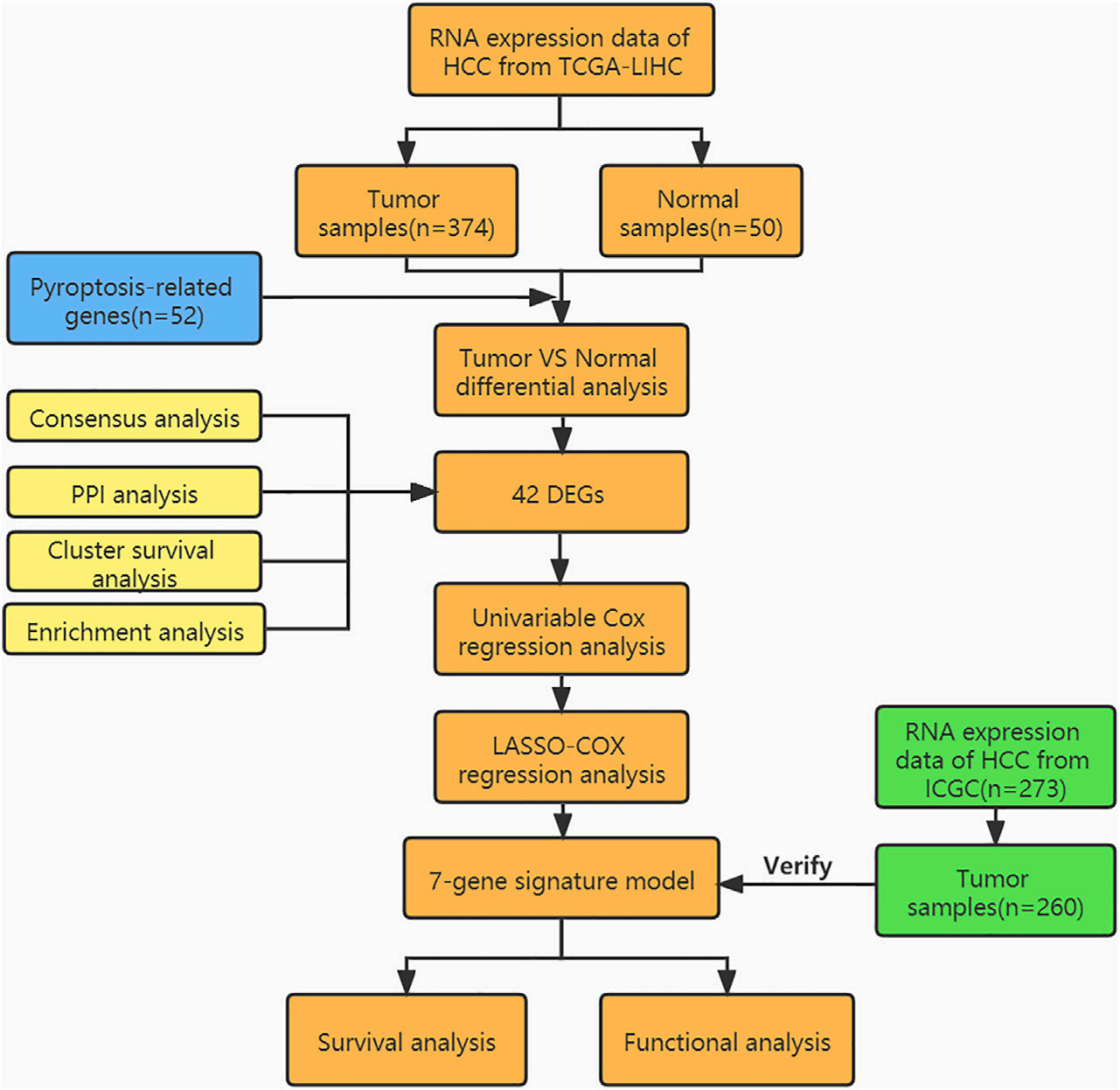
Workflow diagram.

We used Bioconductor packages including “limma,” “survival,” “survminer” in Rstudio software (Version 1.4.1106) for analyzing data. Wilcoxon test and unpaired Student’s t-test were used to comparing non-normal and normal distribution expression variables. Based on the false discovery rate, the different expression of genes was corrected by the Benjamin Hochberg method to control the elevated false-positive rate. Kaplan Meier (KM) survival analysis was performed to evaluate the feasibility of pyroptosis-related genes signature for predicting the overall survival of HCC patients. Time-dependent receiver operator characteristic curve (ROC) and decision curve analysis (DCA) (Vickers et al., 2008) was used to validate the reliability of the predictive model and to compare the accuracy of the novel pyroptosis related gene signature with traditional clinicopathological features in predicting the prognosis of HCC patients. Furthermore, Fisher’s exact test was used to analyze pyroptosis-related gene expression profiles among the clinicopathological features. To analyze the pyroptosis-related DEGs associated immune status in each sample in the TCGA-LIHC cohort, the relative infiltration of 20 immune cell types in the tumor microenvironment was calculated via ssGSEA with the application of the “GSVA” package in R. *p* < 0.05 in the results of all analyses was considered statistically significant. The flow-process diagram of this study is shown in Figure 1.

## Results

### Identification of Pyroptosis-Related DEGs

**FIGURE 2:**
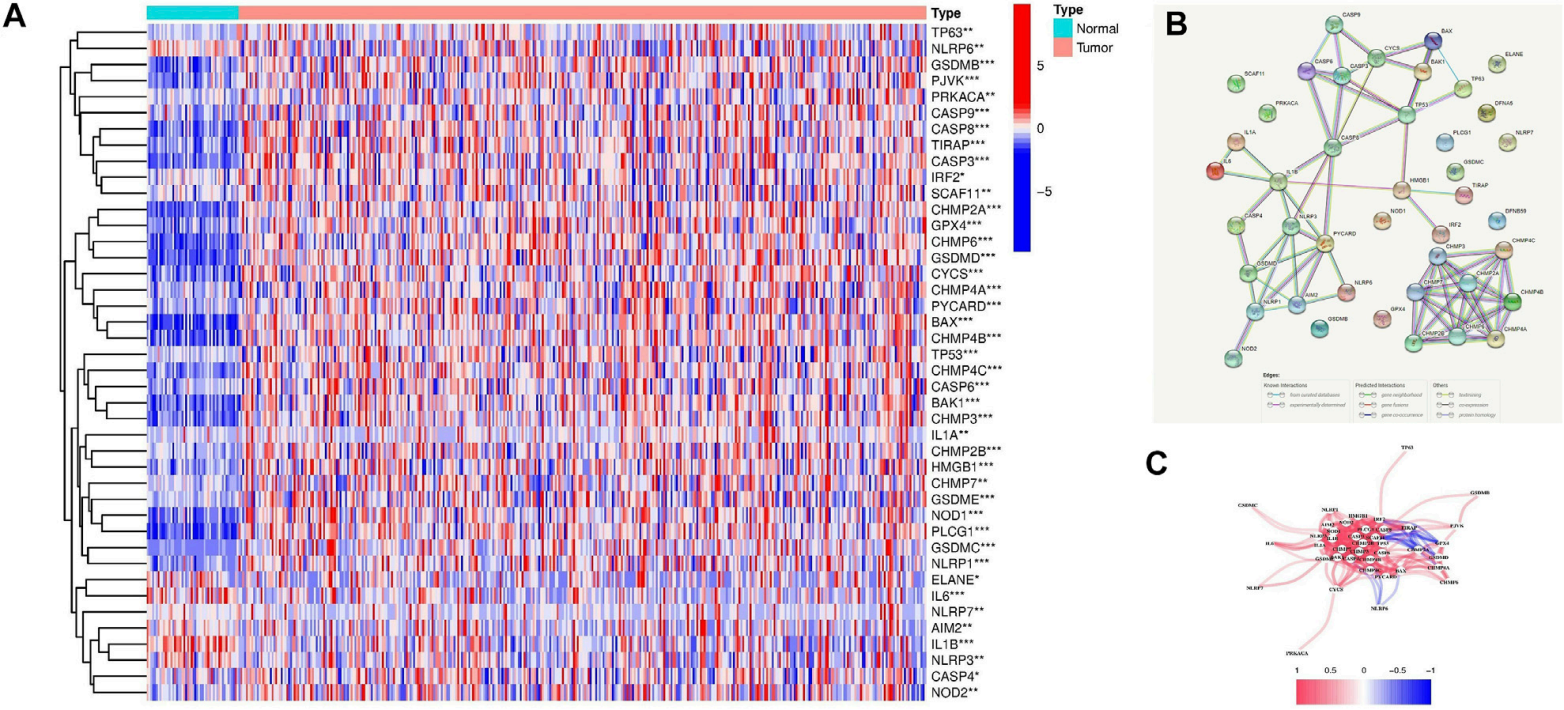
Expression of the 42 pyroptosis-related DEGs and their interactions. **(A)** Heatmap of the pyroptosis-related DEGs between the normal and the tumor samples (blue: low expression level; red: high expression level). *p* values were presented as: *p < 0.05; **p < 0.01; ***p < 0.001. **(B)** The PPI network showed the interactions among the pyroptosis-related DEGs. **(C)** The correlation network of the pyroptosis-related DEGs (blue lines: negative correlations; red lines: positive correlations. The color depth reflected the strength of their relevance).

42 pyroptosis-related DEGs among HCC and normal liver tissues in the TCGA-LIHC dataset were identified using the limma R package (Supplementary Table S3). The expression level of these genes was presented as a heatmap in Figure 2A. Further by PPI analysis, we explored the interactions among these DEGs (Figure 2B). With the minimum required interaction score of 0.9 (the highest confidence) in the PPI analysis, we determined *NLRP3, CHMP4A, CASP8, CASP3, TP53, PYCARD, CHMP2A,* and *IL1B* were hub genes. The correlation network of the pyroptosis-related DEGs is shown in Figure 2C.

### Pyroptosis-Related DEGs-Based HCC Classification Pattern

**FIGURE 3:**
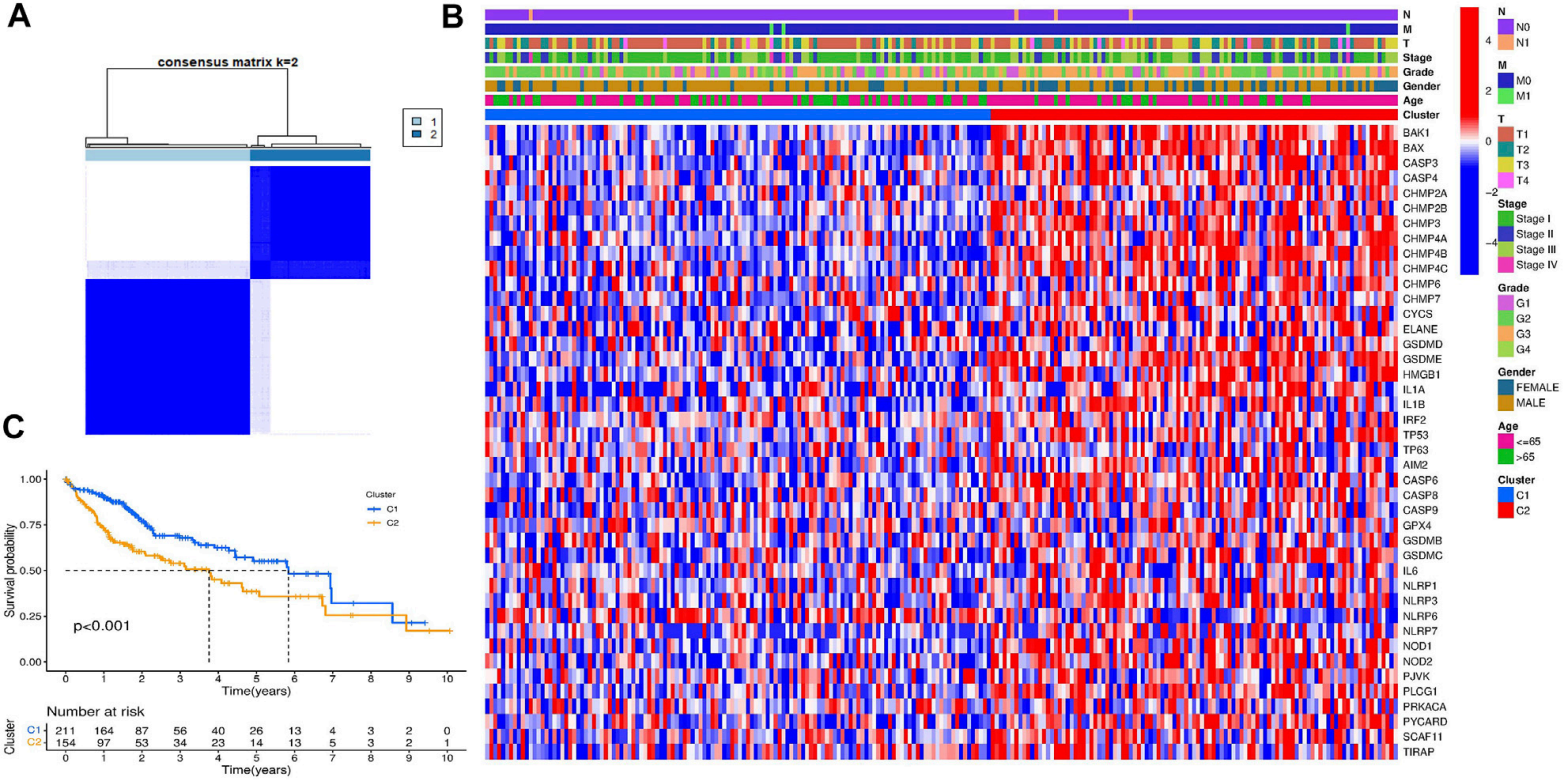
HCC classification pattern based on the pyroptosis-related DEGs. **(A)** 377 HCC patients were divided into two groups when *k* = 2 in the TCGA cohort. **(B)** Heatmap of the clinicopathological characteristics between the two clusters classified by the DEGs. **(C)** KM overall survival curves of the two clusters.

To explore the connections between the expression of the 42 pyroptosis-related DEGs and HCC subtypes, we performed a consensus clustering analysis with all 377 HCC patients in the TCGA-LIHC cohort. By increasing the clustering variable (k) from 2 to 9, we found that when *k* = 2, the intragroup correlations were the highest and the intergroup correlations were low, indicating that the 377 HCC patients could be well divided into two clusters based on the 42 DEGs (Figure 3A). The DEGs expression profile and the clinicopathological characteristics were presented in the heatmap (Figure 3B). We also compared the survival advantage between the two clusters, and the KM overall survival curves showed that the survival probability of cluster 1 was higher than cluster 2 (Figure 3C).

### Enrichment Analysis of Pyroptosis-Related DEGs

**FIGURE 4:**
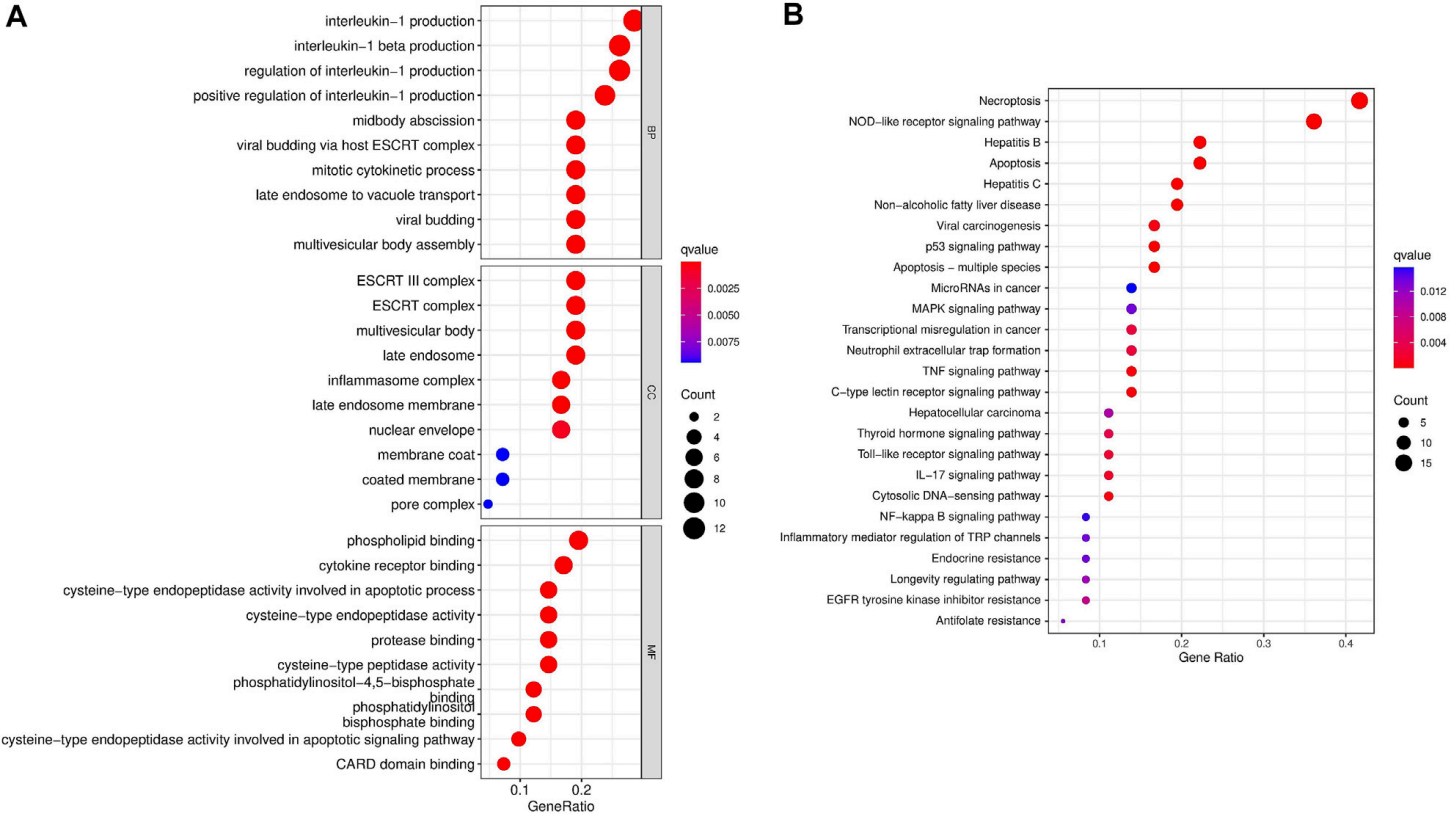
Gene Ontology and KEGG enrichment analysis of the pyroptosis-related DEGs. **(A)** GO analysis. (**B)** KEGG analysis.

Gene Ontology (GO) function and KEGG pathways enrichment analyses of the DEGs were performed. Enriched biological process (BP), including regulation of interleukin−1 production, midbody abscission, and mitotic cytokinetic process. Meanwhile, phospholipid binding, cytokine receptor binding, and cysteine−type endopeptidase activity were the regular molecular function (MF). Cellular component (CC) mainly comprised the ESCRT complex, multivesicular body, late endosome, and inflammasome complex (Figure 4A). Moreover, KEGG pathways analysis demonstrated that necroptosis, NOD−like receptor signaling pathway, apoptosis, hepatitis, P53 signaling pathway, MAPK signaling pathway, and MicroRNAs in cancer were markedly enriched (Figure 4B).

### Development of Pyroptosis-Related Gene Prognostic Signature

**FIGURE 5:**
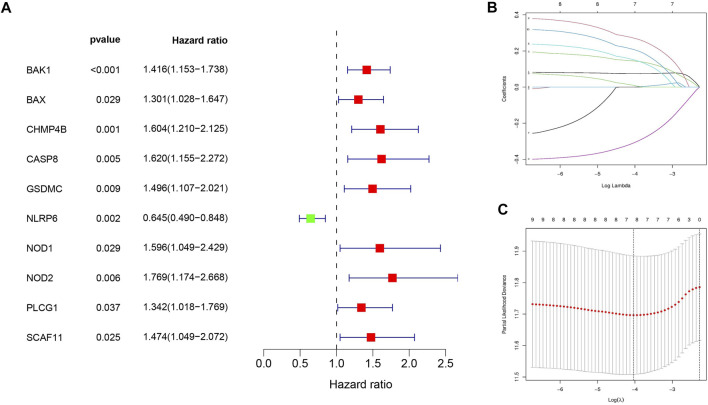
Development of seven pyroptosis-related genes prognostic signature. **(A)** Univariate Cox regression revealed 10 pyroptosis-related genes associated with prognosis. **(B)** 10 pyroptosis-related genes were penalized by LASSO Cox regression analysis. **(C)** 10-fold cross-validation for the optimal parameter selection in the LASSO Cox regression.

First, ten HCC prognosis related pyroptosis genes were screened out from the DEGs by univariate Cox analysis (Figure 5A). Next, the ten pyroptosis-related genes were penalized by LASSO Cox regression (Figures 5B,C). Finally, the pyroptosis-related genes signature was constructed based on the risk score= (0.07486*BAK1 exp.) + (0.14487 *CHMP4B exp.) + (0.15165*GSDMC exp.) + (−0.309234*NLRP6 exp.) + (0.27176 *NOD2 exp.) + (0.00979 *PLCG1 exp.) + (0.20830 *SCAF11 exp.).

### Survival Results and Multivariate External Examination

**FIGURE 6:**
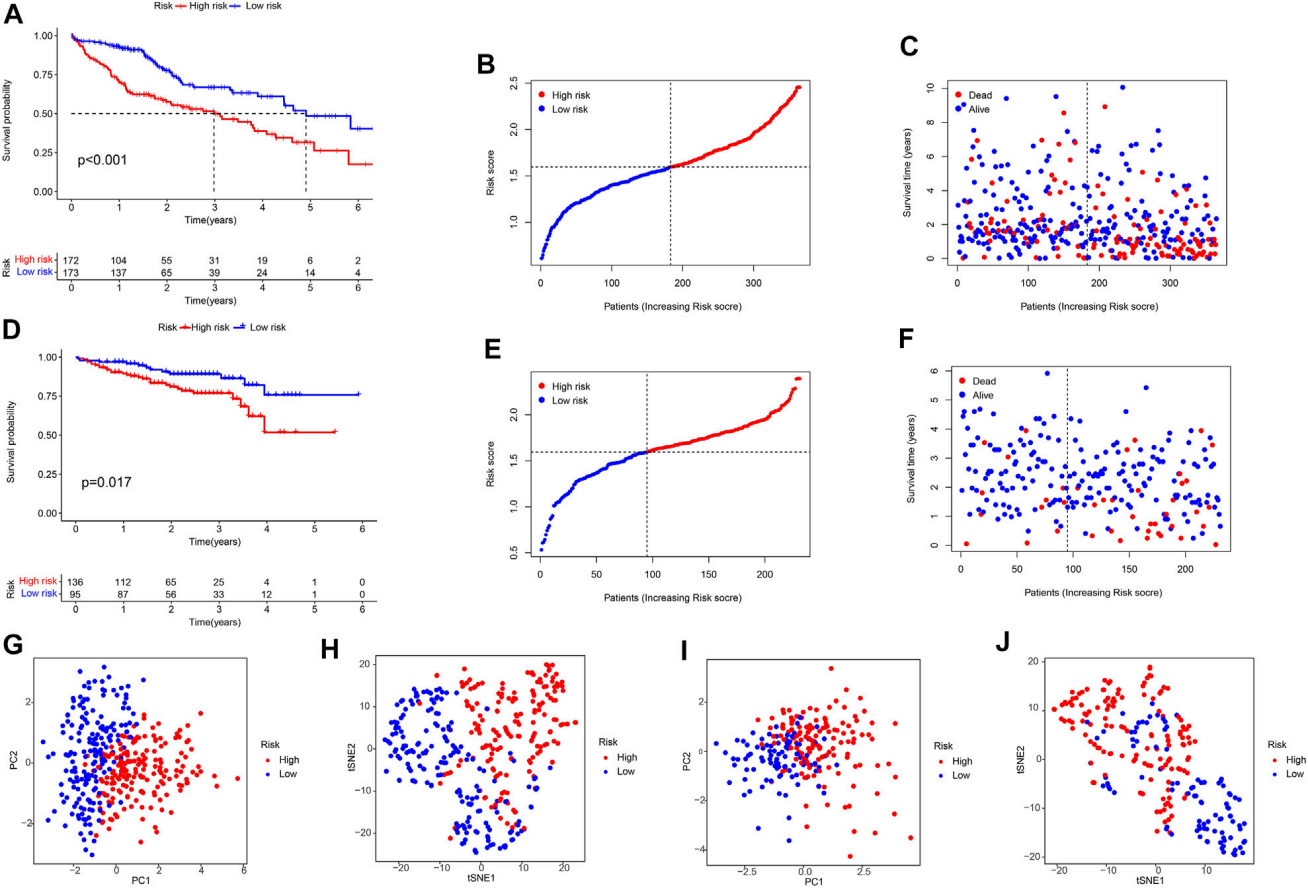
Survival analysis of the seven pyroptosis-related genes signature in the TCGA-LIHC cohort and ICGC-LIRI-JP cohort. TCGA-LIHC cohort **(A–C,G,H)**, ICGC-LIRI-JP cohort **(D–F, I,J)**. **(A,D)** KM survival analysis result. **(B,C,E,F)** Survival status and the risk score distribution of HCC patients. **(G,I)** PCA plot. **(H,J)** t-SNE analysis.

**FIGURE 7:**
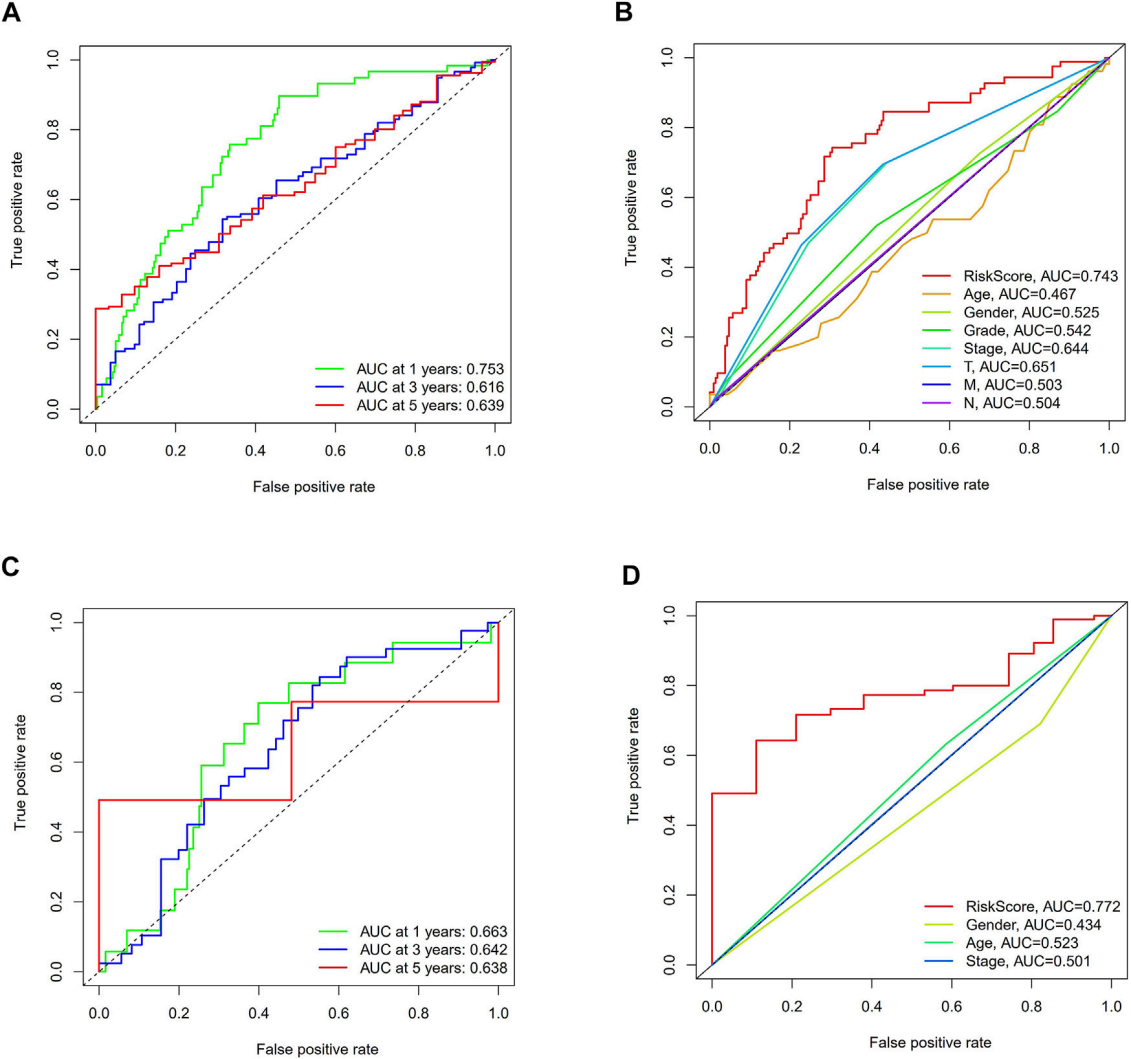
The ROC curve analysis of the seven pyroptosis-related genes signature in the two cohorts. **(A,C)** Time-dependent ROC analysis for HCC patients. **(B, D)** The ROC analysis for clinical features and risk score signature.

KM analysis confirmed that the TCGA and ICGC cohorts HCC patients in the high-risk group were associated with worse OS (Figures 6A,D). At the same time, we could see from the hazard survival status plots of the high-risk groups that high expression of the novel predictive model is correlated with poor survival of HCC patients (Figures 6B–F). Besides, PCA analysis and t-SNE analysis presented that HCC patients in different risk groups were distributed in two directions (Figures 6G–J). Then, we performed ROC analysis using the timeROC package in R. The prognostic prediction power (AUC) of the seven pyroptosis-related genes signature in the TCGA-LIHC cohort was 0.753(1 year), 0.616(3 years), and 0.639 (5 years) (Figure 7A). Furthermore, the AUC of the seven pyroptosis-related genes signature in the IGCG validation cohort was 0.663(1 year), 0.643(3 years), and 0.638 (5 years) (Figure 7C). The clinical characteristics of ROC analysis revealed that compared with the traditional pathological characteristics, the risk score model could more accurately predict the prognosis of HCC patients in the TCGA cohort (AUC = 0.743, Figure 7B) and ICGC cohort (AUC = 0.772, Figure 7D).

### Independent Prognostic Value Validation of the Risk Signature

**FIGURE 8:**
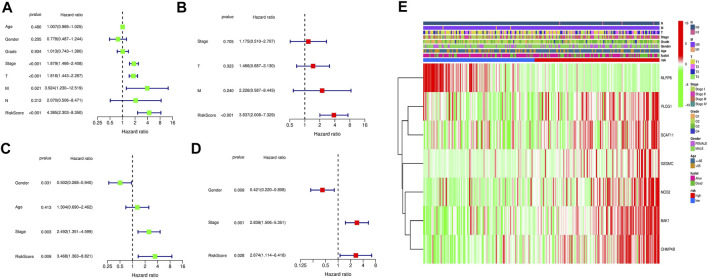
Assessment of the clinical prognostic value of the risk score model in HCC patients by univariate and multivariate COX analyses. **(A)** Univariate independent Cox analysis for TCGA cohort. **(B)** Multivariate independent Cox analysis for TCGA cohort. **(C)** Univariate independent Cox analysis for ICGC cohort. **(D)** Multivariate independent Cox analysis for ICGC cohort. **(E)** Heatmap of the pyroptosis-related genes prognosis signature and clinicopathological manifestations.

**FIGURE 9:**
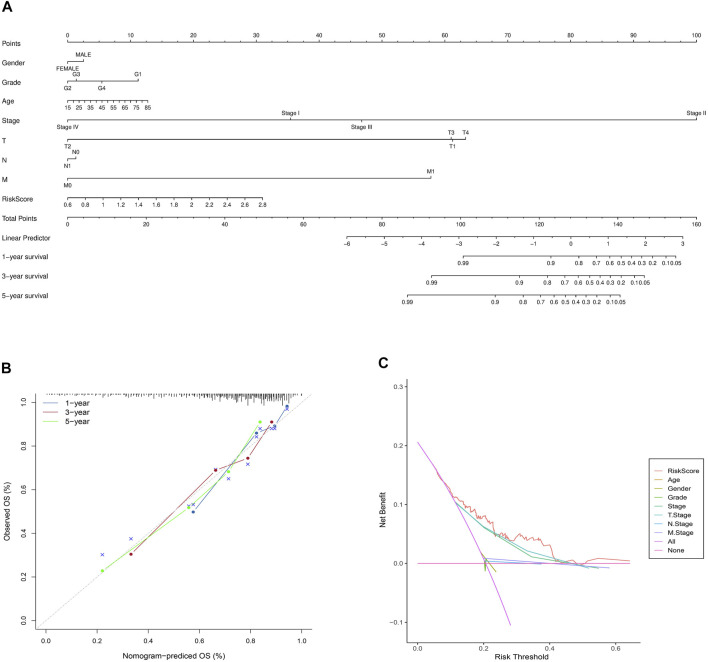
The nomogram model and calibration curves developed based on the risk score signature and prognosis-related clinicopathological indicators. **(A)** The predictive nomogram. **(B)** The calibration curves of the nomogram. **(C)** The decision curve analyses plot.

Univariate and multivariate cox analyses were conducted to verify whether the novel pyroptosis-related genes risk score signature was an independent prognostic factor for overall survival of HCC patients. The risk score model in the TCGA and ICGC cohorts were significantly associated with overall survival of HCC patients in the univariate Cox analysis (TCGA cohort: HR = 4.385, 95% CI = 2.303–8.350, *p* < 0.001; ICGC cohort: HR = 3.468, 95% CI = 1.363–8.821, *p* = 0.009) (Figures 8A,C). After correcting for other confounders, the multivariate Cox analysis confirmed that the risk score signature remained an independent predictor of overall survival for HCC patients. (TCGA cohort: HR = 3.837, 95% CI = 2.008–7.329, *p* < 0.001; ICGC cohort: HR = 2.674, 95% CI = 1.114–6.418, *p* = 0.028) (Figures 8B,D). The clinical heatmap presented the relationship between the novel signature and traditional clinicopathological manifestations in Figure 8E. The fitting degree of calibration curve verified the accuracy of the nomogram model in predicting the prognosis of patients with HCC. (Figures 9A,B). Meanwhile, the net benefit of the risk score signature in the DCA was superior to traditional clinical and pathological characteristics in predicting the prognosis of HCC patients (Figure 9C). Therefore, this nomogram could be used in predicting the prognostic of HCC patients.

### Gene Set Enrichment Analysis

**FIGURE 10:**
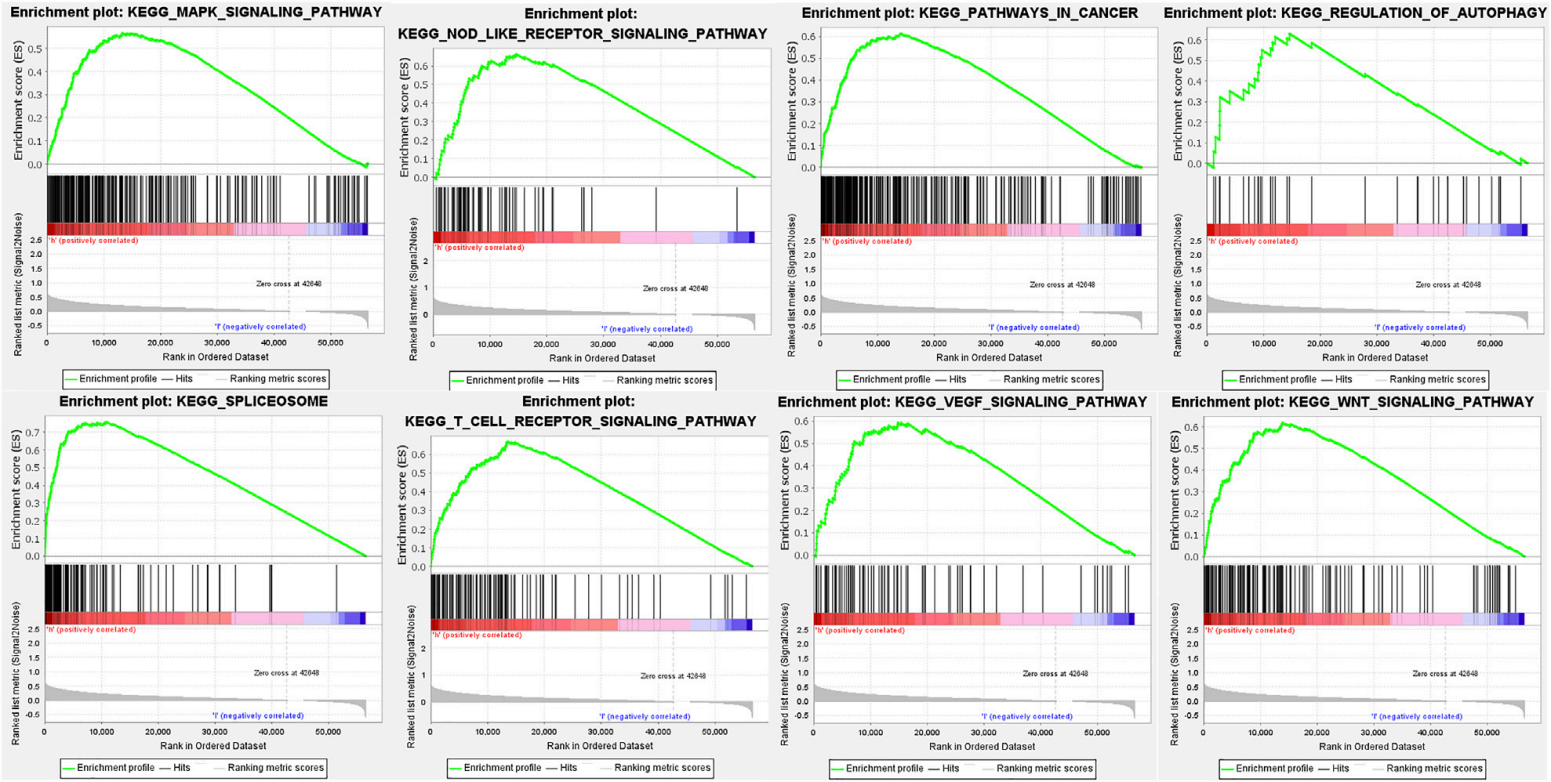
GSEA for the seven pyroptosis-related genes signature.

The potential pathways, mechanisms, and bioprocess of the pyroptosis-related genes signature were analyzed based on GSEA, which revealed those genes regulated both the tumor development and immune response, centrally including NOD-like receptor signaling pathway, T-cell receptor signaling pathway, WNT signaling pathway, regulation of autophagy, MAPK signaling pathway, spliceosome, VEGF signaling pathway and pathways in cancer (Figure 10; Supplementary Table S4).

### Immunological Reaction and Immune Checkpoints Expression

**FIGURE 11:**
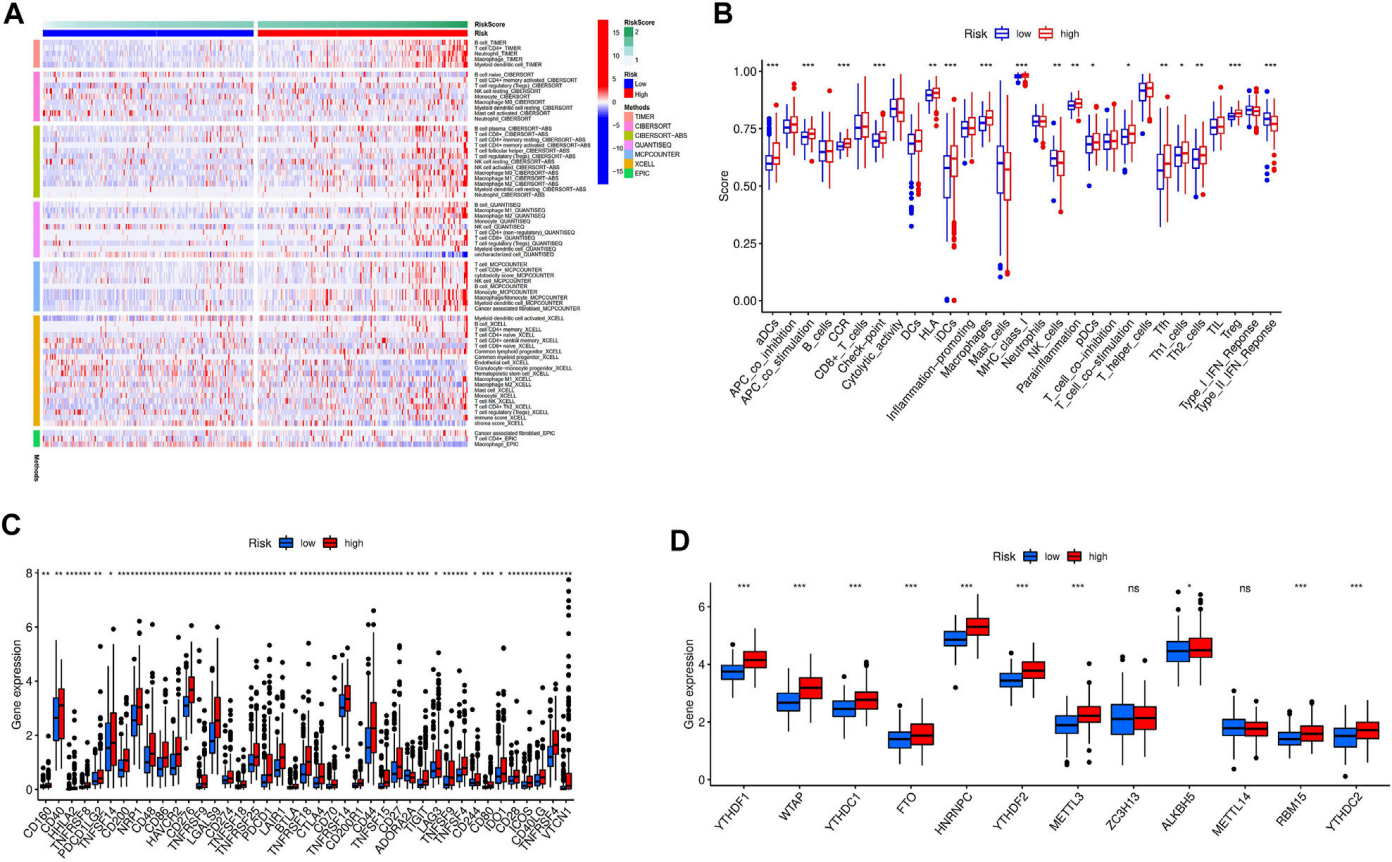
The relationship between prognostic signature and immune response and m6A modification. **(A)** The immune cell infiltration profile of the novel pyroptosis-related genes signature. **(B)** Relevant functional analysis of immune cell subsets. **(C)** Analyses of immune checkpoints between the two HCC risk groups. **(D)** Analyses of m6A modification expression between low and high HCC risk groups.

The Heatmap showed that the expression of the immune cell infiltration responses of the novel pyroptosis-related genes signature was significantly upregulated in HCC under the QUANTISEQ, CIBERSORT, CIBERSORT-ABS, MCPCOUNTER, XCELL, TIMER, and EPIC algorithms (Figure 11A; Supplementary Table S5). Single-sample gene set enrichment analysis based on TCGA-LIHC data showed expression of immune cell subsets and relevant functions, significantly different between the two risk groups. *p* values were presented as: *p < 0.05; **p < 0.01; ***p < 0.001. The high-risk group’s most prominent up-regulated immune functions were aDCs, APC co-stimulation, CCR, check-point, iDCs, Macrophages, MHC class-I, Treg. In contrast, type II INF response was down-regulated in the high-risk group, implying one of the main causes that suppression of the production and release of IFNs leads to loss of control over HCC growth (Figure 11B). Given the importance of immunotherapy based on checkpoint inhibitors for HCC, we further investigated the expressions of immune checkpoints in the two risk groups. The results showed that most immunological checkpoints were more active in high-risk groups in Figure 11C. The analysis of the effect of the pyroptosis-related genes signature on m6A-related modification showed the methylation expression level of *YTHDF1, YTHDF2, WTAP, YTHDC1, YTHDF2, FTO, HNRNPC, ALKBH5, RBM15, YTHDC2,* and *METTL3* in the high-risk group was higher. (Figure 11D).

## Discussion

Cell death is one of the most fundamental problems of life and plays a crucial role in organismal development, homeostasis, and cancer pathogenesis (Hanahan and Weinberg, 2011). As a model of programmed cell death, pyroptosis, although capable of suppressing tumor cell proliferation, can also create a microenvironment suitable for tumor cell growth and promotion (Minton, 2020; Yu et al., 2021), and thus has received increasing attention. Meanwhile, many recent studies have demonstrated that pyroptosis is closely related to developing liver diseases such as liver damage (Lebeaupin et al., 2015), fatty lesions (Miura et al., 2010), inflammation (Wei et al., 2019), and fibrosis (Wree et al., 2014). However, little is currently known about the role of pyroptosis in liver cancer development, and our study was undertaken to elucidate this role. In this study, we first analyzed 42 pyroptosis DEGs in HCC. Based on the pyroptosis -related DEGs, we determined two molecular subtypes using the consensus clustering algorithm. It was found that the survival probability of C2 was much worse than C1 in overall survival. Functional and KEGG pathways analysis further discovered that these DEGs in subtypes primarily participated in necroptosis, NOD−like receptor signaling pathway, apoptosis, hepatitis, P53 signaling pathway, and MAPK signaling pathway. Some recent studies showed that Caspase/granzyme-induced apoptosis could be switched to pyroptosis by the expression of GSDMs, appears to contribute to the killing of tumor cells by cytotoxic lymphocytes, and reprogram the tumor microenvironment to an immunostimulatory state (Van Opdenbosch and Lamkanfi, 2019; Tsuchiya, 2020; Tsuchiya, 2021). Zhang et al. (2019) reported that overexpression of p53 in human lung cancer alveolar basal epithelial cells significantly reduced tumor growth and mortality by increasing pyroptotic levels in an *in vivo* assay. Therefore, appropriate guiding the pyroptosis of hepatocellular carcinoma cells may inspire an advanced therapy strategy of HCC patients.

Next, our study identified seven differently expressed pyroptosis-related gene markers from DEGs as independent prognostic factors for HCC. Among the seven pyroptosis-related genes signature, *BAK1* is a vital cell death regulator that can initiate mitochondria-mediated apoptosis by interacting with proteins (Wang et al., 2013). The protection of *BAK1* by exosomal circ-0051443 through sponging mir-331-3p can inhibit the malignant biological behaviors of HCC(Chen et al., 2020). And silencing CHMP4B can promote epithelial-mesenchymal transition in HCC(Han et al., 2019). *GSDMC* is the only one of the human gasdermin family members whose biological function has not been determined (Kovacs and Miao, 2017). *GSDMC* was significantly associated with poorer prognosis liver cancer patients in our study, indicating that it acts as a tumor-promoting gene. Interestingly, the current study revealed that TNF *α* - activated caspase-8 switched apoptosis to pyroptosis in the presence of hypoxia-activated *GSDMC* and nPD-L1, leading to tumor necrosis in hypoxic regions (Hou et al., 2020; Du et al., 2021). Therefore, the effect of activating *GSDMC* in different environments on liver cancer is worthy of further exploration. Wang Q et al. (2018) reported that *NLRP6* inhibits gastric cancer cell proliferation, migration, and invasion by regulating the STAT3 signaling pathway, and its down-regulation is closely associated with poor patient prognosis. Similarly, down-regulation of *NLRP6* was associated with poorer prognosis in HCC patients in our study, suggesting that *NLRP6* may play a tumor suppressor role in HCC development. Meanwhile, hepatic *NOD2* promotes hepatocarcinogenesis through a *RIP2* mediated proinflammatory response and novel nuclear autophagy-mediated DNA damage mechanism, and its high expression is closely associated with poor prognosis in HCC patients (Zhou et al., 2021). Furthermore, increased *PLCG1* expression in tumor tissues was significantly associated with adverse clinical features of HCC, which may be a role played by *PLCG1* through activation of mitogen-activated protein kinase and NF-kB signaling pathways (Tang et al., 2019). To date, there are few studies on the regulation of pyroptosis by *SCAF11* in cancer (Xu et al., 2021; Ye et al., 2021). In our study, high expression of *SCAF11* was associated with poor prognosis in liver cancer, reflecting that it may be a liver cancer-promoting factor associated with positively regulating the pyroptosis pathway and inhibition of *SCAF11* should be considered as a target for the treatment of HCC. Based on the median value of the risk score of pyroptosis-related genes signature, HCC patients were divided into high-risk and low-risk group. The survival analyses indicated that the pyroptosis-related high-risk genes were positively related with worse prognosis HCC patients. Moreover, the pyroptosis-related genes signature performed well in the ROC and DCA validation. Finally, their reliability and applicability in predicting HCC prognosis were demonstrated in the nomogram and calibration curve and indicated that our novel risk signature outperformed traditional clinicopathological characteristics.

Pyroptosis serves as a bridge between the immune system and the tumor (Li et al., 2021). Its activation in immune cells and cancer cells will cause the release of inflammatory chemokines and subsequent immune cell infiltration, activating the tumor microenvironment and improving the tumor’s efficiency of immunotherapy (Xia et al., 2019; Vietri et al., 2020). On the other hand, the chronic inflammatory response resulting from pyroptosis triggered inflammasomes, and produced cytokines can help tumor cells escape from immune system surveillance and promote the development of tumors (Cookson and Brennan, 2001; Wang Q et al., 2020). In GSEA analysis, the significant enrichment of immune and tumor-related pathways among individuals in the high-risk group indicated two sides of the effect of pyroptosis on tumor cell survival, progression, and apoptosis. Furthermore, relevant functional analysis of immune cell subsets revealed that aDCs, APC co-stimulation, CCR, check-point, iDCs, Macrophages, MHC class-I, and Treg of pyroptosis-related genes signature were significantly attenuated in HCC high-risk group, suggesting that reduced levels of antitumor immunity may lead to poor prognosis. Therefore, promoting antitumor immune response is essential to prevent HCC at early stage from further development and generate effective clinical treatments. Moreover, the expression of Immune checkpoints such as *PDCD1*, *PDCDLG2*, *TIGIT*, *LAG3*, and *TNFRSF4* was enhanced in the high-risk group. The PD-1 pathway is a central pathway of immunosuppression in the human tumor microenvironment. Inhibition of PD-1 and PD-L1 can generate endogenous antitumor immunity to inhibit cancer development (Garg and Agostinis, 2017). However, the response rate may be low since inflammation in the cancer-immune microenvironment is ineffective for efficient infiltration and activation of immune cells. The efficiency of anti-PD-1 or PD-L1 therapy can be improved under pyroptosis-induced inflammation in the tumor microenvironment by chemotherapy, radiotherapy, and other therapeutic regimens (Bergsbaken et al., 2009; Reck et al., 2019). Published clinical trials have shown that antibiotic chemotherapeutics can promote the combination of STAT3 and PD-L1 to upregulate *GSDMC* mediated pyroptosis under hypoxia (Blasco and Gomis, 2020), which may improve HCC patient survival compared to patients received only a single type of treatment to improve the efficiency of PD-L1 inhibitors. TIGIT, similar to LAG3, belongs to the immunoglobulin superfamily and is exclusively expressed on lymphocytes, including CD8 + T cells, memory, and regulatory CD4 + T cells, follicular CD4 + T cells, and NK cells (Stanietsky et al., 2009; Ge et al., 2021). In HCC tumor-bearing mice treated with anti-PD-1, concurrent anti-TIGIT treatment resulted in a combined blockade effect that expanded the effector memory CD8 + T cell population and increased the cytotoxic T cell to Treg ratio in the tumor, thereby suppressing tumor growth and prolonging survival (Li et al., 2018; Chiu et al., 2020; Lepletier et al., 2020), indicating that TIGIT can be used as a rational target to further improve the efficacy of anti-PD-1 therapy in HCC. Unlike standard immune checkpoint blockers that block surface receptors in tumors and T cells responsible for inhibiting antitumor immune responses, drugs that target *TNFRSF4* work by directly activating and modulating the immune response (Alves Costa Silva et al., 2020). Upon treatment of tumor models with an anti-*TNFRSF4* monoclonal antibody, IL-10 production by tumor-infiltrating Treg cells is reduced, allowing the maturation of dendritic cells (Burocchi et al., 2011; Zhang et al., 2018), creating a permissive immune state that allows for the maturation of dendritic accumulation of myeloid cells and development of innate and adaptive immunity (Piconese et al., 2008; Bulliard et al., 2014), opening an additional avenue for cancer therapy.

Although we verified two subtypes of HCC and validated the reliability of the novel predictive risk score model of seven pyroptosis genes and analyzed their functions in HCC progression, our study has serval limitations. This bioinformatic study needs to be tested further by experimental validation. Therefore, further laboratory experiments are required, including larger sample multicenter studies, especially studying the relationship between pyroptosis-related genes signature and immune activity. Compared with other traditional clinical characteristics, our risk score model is a better independent prognostic indicator. Thus, this novel risk model could serve as the prognostic predictor and provide clues for personalized immunotherapy for HCC patients.

## Conclusion

The novel pyroptosis-related genes signature can predict the prognosis of patients with HCC and insight into new cell death targeted therapies.

## Data Availability

The original contributions presented in the study are included in the article/Supplementary Material, further inquiries can be directed to the corresponding authors.
